# Breast Cancer Characteristics after Metabolic and Bariatric Surgery: A Matched Comparison to Patients with Severe Obesity

**DOI:** 10.1007/s11695-025-07916-3

**Published:** 2025-05-27

**Authors:** Jawad Tome, Marian Khatib, Eran Nizri, Lilah Margalit Grigg, Lior Orbach, Guy Lahat, Shai Meron Eldar, Adam Abu-Abeid

**Affiliations:** 1 Division of Surgery, Tel Aviv Sourasky Medical Center, Tel Aviv, Israel; 2 Faculty of Medical and Health Sciences, Tel Aviv University, Tel Aviv, Israel

**Keywords:** Bariatric surgery, Metabolic surgery, Severe obesity, Weight loss, Breast cancer, Survival

## Abstract

**Background:**

Severe obesity increases breast cancer (BC) risk and progression. Metabolic and Bariatric Surgery (MBS) modulates metabolic and hormonal pathways, potentially influencing cancer biology. This study evaluates BC patients after MBS. The aim of this study is to assess the impact of MBS on BC, focusing on disease-free survival (DFS), presentation, subtypes, and oncologic outcomes.

**Methods:**

A retrospective analysis of a single-center database included patients undergoing BC surgery after MBS (2012–2020), matched (1:4) to patients with severe obesity undergoing BC surgery.

**Results:**

Among 696 patients, 29 (4%) had BC post-MBS. Sleeve gastrectomy was the most common procedure (48.2%). Mean age at BC surgery was 60.7 ± 10 years. BMI prior to BC surgery was lower in the MBS-group (32.4 vs. 38.3 kg/m^2^, *p* < 0.0001). Disease-free survival (114 vs. 146 months, *p* = 0.75) and recurrence rates were similar. The MBS-group had lower luminal-A subtype rates (34.4% vs. 59.5%, *p* = 0.01) and higher luminal-B subtype rates (58.6% vs. 27.6%, *p* = 0.001). No patients in the MBS-group had ductal carcinoma in situ (DCIS) (0% vs. 20%, *p* = 0.03). Other subtypes showed no differences.

**Conclusion:**

MBS may influence BC pathogenesis, with lower DCIS and luminal-A rates. These findings suggest a potential reduction in overall BC incidence due to metabolic and hormonal changes after MBS. Oncologic outcomes remained comparable.

**Graphical Abstract:**

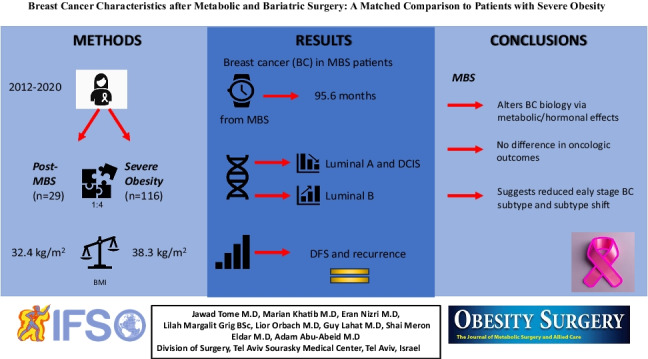

## Introduction

Breast cancer (BC) is the most commonly diagnosed cancer globally and is the fourth most common cause of cancer deaths worldwide [1]. The burden related to BC is expected to increase to over 3 million cases annually in 2040 [2]. There are many reasons to the increase in BC rates including an alteration in risk factor profile, better cancer registration, and BC detection [3].

Severe obesity is a modifiable risk factor associated with at least 14 types of cancers, including BC. This link is likely driven by obesity-related changes in insulin pathways and sex hormone levels [4]. The rate of obesity and severe obesity is constantly increasing and affects approximately 40.3% and 9.4% of adults in the USA, respectively [5].

The most effective treatment for severe obesity is metabolic and bariatric surgery (MBS). Many studies have shown satisfactory results in terms of durable weight loss, resolution of obesity-related diseases such as type 2 diabetes (T2D), and reduction in certain cancer rates [6–8]. MBS has been reported to reduce the risk of BC in patients with severe obesity [4, 9]. Despite this, BC after MBS is still encountered during clinical practice. The characteristics of BC in patients after MBS is unclear and could differ from non-MBS patients.

The purpose of this study is to determine the outcomes of MBS on patients diagnosed with BC. The primary outcome was to evaluate disease-free survival (DFS). Secondary outcomes included breast cancer presentation, subtypes, and other oncologic outcomes.

## Methods

### Patients

This is a retrospective analysis of a prospectively maintained database in a single tertiary center. All adult patients (≥ 18 years old) undergoing BC surgery after MBS were included (MBS-group). The study group was matched to a control group (1:4) of patients with body mass index (BMI) ≥ 35 kg/m^2^ undergoing breast surgery due to BC (non-MBS-group). The matching was conducted based on age. Patients undergoing BC surgery younger than 18 years old and pregnant women were excluded.

### Baseline Characteristics

Data captured included patients’ baseline characteristics — age, gender, pre-MBS BMI, BMI prior to BC surgery, and total weight loss (TWL).

### BC Characteristics

Data regarding risk factors of BC was also withdrawn including family history of BC, history of assisted reproductive technology (ART), number of pregnancies, number of deliveries, hormonal replacement treatment (HRT), and *BRCA* mutations. Additional data captured included tumor-related characteristics — way of presentation (mass palpation/screening), preoperative BC pathology, neoadjuvant chemotherapy, postoperative BC pathology, the change in BC pathology postoperatively, margin involvement, breast cancer subtypes according to receptor status (Luminal A, Luminal B, triple negative, *Her2* enriched), tumor grade, and lymph node (LN) status.

### Perioperative Outcomes

We analyzed the data in regard to perioperative outcomes including type of BC surgery (lumpectomy/mastectomy), LN resection type (sentinel LN biopsy/axillary LN dissection), duration of surgery, hospital length of stay and major 30-day complications.

### Oncologic Outcomes

Following surgery data was withdrawn from patient follow-up, and we analyzed DFS and recurrence rates.

### Statistical Analysis

Data are presented as mean and standard deviation or as number and its correspondent percentage. The association between two qualitative variables was assessed using the chi-square test. Differences between groups were assessed by independent-samples *t* test. Survival analysis was performed according to the Kaplan–Meier method, and survival curves were compared using the log-rank test. A *p* value below 0.05 was considered significant. Statistical analysis was performed using SPSS (version 29) software, and graphics were obtained using GraphPad.

## Results

In the corresponding study period, a total of 29 patients underwent BC surgery following MBS (MBS-group), and 696 patients with BMI ≥ 35 kg/m^2^ underwent BC surgery. The matched control group was set and included 116 patients (non-MBS group).

### MBS-Related Data

Sleeve gastrectomy (SG) was the most prevalent MBS (*n* = 14, 48.2%), followed by adjustable gastric band (AGB) (*n* = 8, 27.5%), Roux-en-Y gastric bypass (*n* = 3, 10.3%), vertical banded gastroplasty (*n* = 3, 10.3%), and one anastomosis gastric bypass (*n* = 1, 3%). The mean pre-MBS BMI was 42.6 ± 7 kg/m^2^. All patients had no major MBS complications either within 30 days or during follow-up. Two patients underwent conversion from AGB to SG due to weight regain. The mean BMI at BC surgery was 32.4 ± 6.5 kg/m^2^ with a mean TWL of 25.5% ± 13.5 The median time interval from MBS to BC surgery was 95.6 months. All included patients were diagnosed with and presented with BC following their MBS.

### Comparative Outcomes

The baseline characteristics of the cohort are shown in Table 1 — There were 142 women (98%) in the cohort and the mean age was 60.7 ± 10 years with no statistically significant difference between groups. The BMI at the time of BC surgery was significantly lower in the MBS-group when compared to the non-MBS group (32.4 ± 6.5 kg/m^2^ versus 38.6 ± 4.1 kg/m^2^, *p* < 0.0001). There was no significant difference between groups in risk factors of BC including family history of BC, *BRCA* mutations, obstetrical history, ART treatments, and HRT.

**Table 1:** Baseline characteristics of the study population

		MBS group *n* = 29	Non-MBS group *n* = 116	*P* value
Age, mean in years, SD		59.6, SD = 9.9	61.0, SD = 10.06	0.486
BMI, mean in kg/m^2^, SD	At MBS At BS	42.6, SD = 7.01 32.4, SD = 6.5	38.3, SD = 4.1	< 0.001
Gender, female/male		28:1	114:2	0.56
Family history of BC, *n* (%)	Yes No	16 (55.1%) 13 (44.9%)	35 (30.1%) 81 (69.9%)	0.134
History of HRT, *n* (%)	Yes No	5 (17.2%) 24 (82.8%)	12 (10.3%) 104 (89.7%)	0.302
BRCA mutation, *n* (%)	Yes No	1 (3.4%) 28 (96.6%)	4 (3.4%) 112 (96.6%)	1
Past ART, *n* (%)	Yes No	1 (3.4%) 28 (96.7%)	6 (5.1%) 110 (94.9%)	0.69
Number of pregnancies, mean, SD		2.61, SD = 1.75	2.32, SD = 1.55	0.394
Number of deliveries, mean, SD		2.57, SD = 1.73	2.14, SD = 1.47	0.185

*MBS* metabolic bariatric surgery, *n* number, *BMI* body mass index, *SD* standard deviation, *BC* breast cancer, *HRT* hormone replacement therapy, *ART* assisted reproductive technology

The characteristics of BC are shown in Table 2 — The method of BC diagnosis (screening/mass palpation) was insignificantly different between groups. There was no difference in BC pathology pre- and postoperatively except for ductal carcinoma in situ (DCIS) which was significantly higher in the non-MBS group (20% versus 0%, *p* = 0.03). When evaluating the receptor profiles of BC, patients in the MBS group had significantly less Luminal-A type (34.4% versus 59.4%, *p* = 0.01) and had significantly higher Luminal-B type (58.6% versus 27.6%, *p* = 0.001). The non-MBS group was more likely to undergo preoperative LN biopsy (76.7% vs 55%, *p* = 0.02); however, there was no significant difference between groups in the biopsy results.

**Table 2:** Oncologic characteristics of breast cancer

		MBS group *n* = 29	Non-MBS group *n* = 116	*P* value
What led to the diagnosis? *n* (%)	Screening Symptoms/palpation	16 (55.1%) 13 (44.9%)	72 (62.0%) 44 (38.0%)	0.49
Pathology on biopsy of the primary tumor, *n* (%)	IDC ILC DCIS ADH Papilloma	25 (86.2%) 4 (3.8%) 0 0 0	83 (71.5%) 10 (8.6%) 20 (17.2%) 2 (1.7%) 1 (0.8%)	0.1 0.17 **0.01** 0.47 0.61
Pathology after resection, *n* (%)	IDC ILC DCIS CR	23 (79.3%) 4 (13.7%) 0 2 (6.9%)	78 (67.2%) 9 (7.7%) 23 (20%) 6 (5.1%)	0.20 0.31 **0.008** 0.71
Was there a change in pathology? *n* (%)	Identical Downgraded Upgraded CR	27 (93.1%) 0 0 2 (6.9%)	98 (84.4%) 6 (5.2%) 6 (5.2%) 6 (5.2%)	0.23 0.21 0.21 0.71
Cancer types — according to receptor status, *n* (%)	Luminal A Luminal B TNBC HER2 enriched	10 (34.4%) 17 (58.6%) 2 (7%) 0	69 (59.5%) 32 (27.6%) 12 (10.3%) 3 (2.6%)	**0.01** **0.001** 0.57 0.38
Pathological T stage, *n* (%)	In situ 1 2 4 CR Unknown	0 18 (62%) 9 (31%) 0 2 (7%) 0	23 (20%) 58 (50%) 26 (22.4%) 1 (0.8%) 6 (5.2%) 2 (1.6%)	**0.03** 0.24 0.33 0.61 0.71 0.47
Need for preoperative LN biopsy, *n* (%)	Yes No	13 (44.8%) 16 (55.2%)	27 (23.3%) 89 (76.7%)	**0.035**
Clinical positive LN, *n* (%)^a^	No Yes	21 (72.4%) 8 (27.6%)	97 (83.6%) 19 (16.4%)	0.166
Pathologic N stage, *n* (%)	0 1 2 3 No Resection	20 (68.9%) 8 (27.5%) 1 (3%) 0 0	65 (56%) 20 (17.2%) 8 (6.9%) 3 (2.6%) 20 (17.2%)	0.2 0.2 0.14 0.38 **0.01**
Grade, *n* (%)	1 2 3 Unknown	2 (6.8%) 15 (51.7%) 8 (27.6%) 4 (13.7%)	9 (7.7%) 48 (41.4%) 36 (31%) 23 (19.9%)	0.87 0.31 0.72 0.45
Margins involvement, *n* (%)	Involved Uninvolved	5 (17.2%) 24 (82.8%)	8 (6.9%) 108 (93.1%)	**0.08**
Mucinous subtype, *n* (%)	Yes No	3 (10.3%) 26 (89.6%)	6 (5%) 109 (93.9%)	0.308
Neoadjuvant treatment, *n* (%)	Yes No	8 (27.5%) 21 (72.5%)	21 (18.1%) 95 (91.9%)	0.254

*MBS* metabolic bariatric surgery, *n* number, *SD* standard deviation, *IDC* invasive ductal carcinoma, *ILC* invasive lobular carcinoma, *DCIS* ductal carcinoma in situ, *ADH* atypical ductal hyperplasia, *CR* complete response, *TBNC* triple negative breast cancer, *LN* lymph node

^a^Lymph node metastasis that was palpable on physical examination, imaging, and positive for malignancy on pre-operative biopsy

The perioperative outcomes are depicted in Table 3 — There was no significant difference in most parameters — type of resection (breast-conserving versus mastectomy), oncoplastic surgery, duration of surgery, and hospital length of stay. No major complications were recorded. Although unsignificant, the MBS group had a higher rate of sentinel lymph node biopsy (79.2% vs 63%, *p* = 0.09) with no difference in axillary lymph node dissection rates.

**Table 3:** Operative and postoperative outcomes

		MBS group *n* = 29	Non-MBS group *n* = 116	*P* value
Type of resection, *n* (%)	BCS Mastectomy	26 (89.7%) 3 (10.3%)	103 (88.8%) 13 (11.2%)	0.89
Lymph node resection type, *n* (%)	No LN Resection SLNB ALND	0 23 (79.2%) 6 (20.7%)	22 (18.9%) 73 (63%) 21 (18.1%)	**0.01** 0.09 0.75
Plastic surgery, *n* (%)	Yes No	4 (13.8%) 25 (86.2%)	15 (13%) 101 (87%)	0.9
Duration, min, SD		156.4, SD = 89.8	157.9, SD = 68.2	0.93
Hospital stay in days, SD		1.7, SD = 2.5	2.1, SD = 2.1	0.39
Recurrence incidence, *n* (%)	Yes No	3 (10.3%) 26 (89.7%)	14 (12%) 102 (88%)	0.79
Type of recurrence, *n* (%)	Locoregional Systemic No recurrence	2 (6.9%) 1 (3.4%) 26 (89.7%)	11 (9.5%) 3 (2.5%) 102 (88%)	0.66 0.8 0.79

*MBS* metabolic bariatric surgery, *SD* standard deviation, *n* number, *BCS* breast conserving surgery, min, minutes, *SLNB* sentinel lymph node biopsy, *ALND* axillary lymph node dissection

**Fig. 1:**
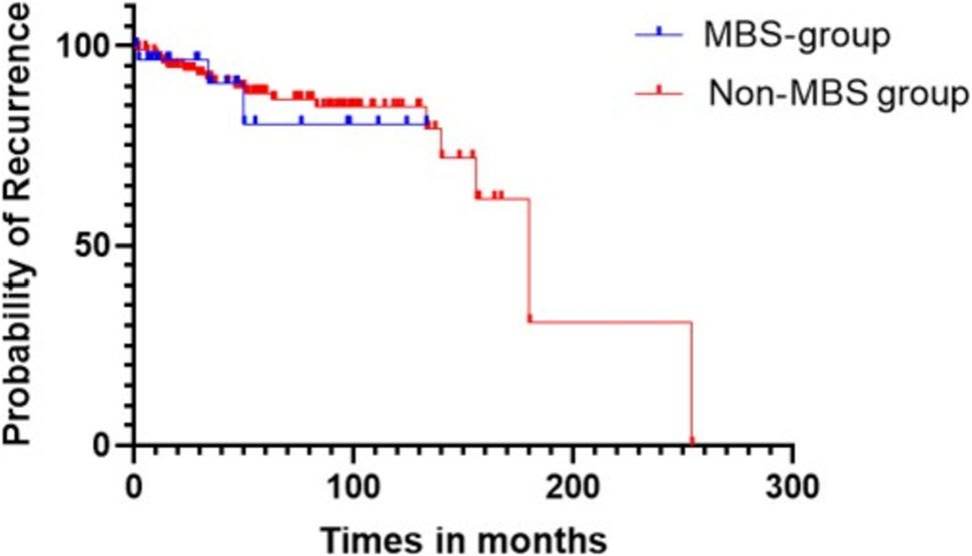
DFS, disease-free survival. Kaplan–Meier graph

The DFS outcomes are shown in Fig. 1 — There was no statistically significant difference between groups in DFS (MBS 114 months versus non-MBS 146.6 months, *p* = 0.75), local recurrence rates (MBS 6.8% versus non-MBS 9.5%, *p* = 0.66), and systemic recurrence rates (MBS 3.4% versus non-MBS 2.5%, *p* = 0.80). During follow-up, there were 15 deaths in the entire cohort with no significant difference between MBS and non-MBS group (2 vs 13; *p* = 0.49). The reason for death was metastatic BC in five patients.

## Discussion

In this retrospective comparative study, we analyzed BC characteristics in patients after MBS compared with patients with severe obesity who did not undergo MBS. The median time interval from MBS to BC surgery was 95.6 months. As expected, there was a significant difference in BMI at the time of BC surgery, with the MBS group presenting with a markedly lower BMI. The study showed that patients in the MBS group had significantly lower rates of common BC pathologies such as DCIS and Luminal-A type. On the other hand, less common pathologies such as Luminal-B type were higher. In terms of oncologic outcomes, there was no difference between groups. Margin involvement was nearly significant (*p* = 0.08) with a relatively higher rate of margin involvement in the MBS group. We believe this likely lacks clinical significance and may be due to the cohort’s limited sample size.

The benefit of MBS on cancer development has been well documented. Adams et al. [10] retrospectively evaluated 21,387 MBS patients and compared to a matched group. MBS patients had had a 25% lower risk of developing any type of cancer, and the incidence was even lower among females — 41% lower risk of cancer development and lower cancer-related mortality. Similarly, Aminian et al. [11] performed a matched cohort study of patients undergoing MBS to a control group of patients with severe obesity. The primary endpoint of the study was the first occurrence of 1/13 types of obesity-related cancers. They found that the cumulative incidence of the primary end point at 10 years was 2.9% in the MBS group and 4.9% in the non-MBS group. When addressing cancer-related mortality at 10 years, they found the cumulative incidence to be 0.8% in the MBS group and 1.4% in the non-MBS group. The results of these large cohort studies suggest that MBS may provide protective benefits beyond metabolic outcomes, potentially lowering long-term cancer risk and cancer-related mortality in patients with severe obesity.

BC remains the most diagnosed cancer in women globally with 1.7 million women diagnosed annually [12]. The link between obesity and BC has been attributed to several biological mechanisms, particularly in postmenopausal women. Excess adipose tissue leads to increased peripheral aromatization of androgens to estrogens, elevated circulating insulin, and reduced sex hormone–binding globulin. This imbalance is believed to contribute to the increased risk of BC in postmenopausal women with severe obesity [13]. Hyperinsulinemia, which is commonly seen in patients with type 2 diabetes, has been reported to be an independent risk factor for BC and plays a significant role in the BC evolution in patients with severe obesity [14]. The mechanism in which insulin increases BC risk is complex and includes increased cell mitosis, decreased apoptosis, decrease in sex hormone–binding globulin which results in increased estrogen. In fact, BC cells commonly overexpress insulin receptors [9]. Kristensson et al. [9] reported the outcomes of a nonrandomized controlled trial representing the Swedish Obesity Subjects study. They found that women after MBS had a reduced risk of BC when compared to conservative management of obesity after 23.9 years. Interestingly, they found that this benefit was mainly observed in patients with preoperative hyperinsulinemia suggesting insulin to be a predictor of treatment. Leptin, another adipokine elevated in obesity, is known to promote angiogenesis and cell proliferation in BC and may contribute to more aggressive tumor behavior [15].

Obesity is also associated with more aggressive clinicopathological features of BC. Proskuriakova et al. retrospectively analyzed 927 patients with BC and found a significant association of severe obesity with tumor size and lymph node involvement [16]. Ayoub et al. reported similar results in postmenopausal women with severe obesity showing increased stage, grade, and worse prognostic features at presentation. There was no difference in receptor profile between groups. In addition, BMI was a predictor for local and distant recurrence of BC [17]. Furthermore, a Canadian meta-analysis including 11 studies with a total of 1,106,939 participants reported a lower overall cancer diagnosis rate in the MBS group (0.54%) compared to controls (0.84%), with a risk ratio (RR) of 0.5. MBS was associated with a higher likelihood of detecting stage I cancer and a reduced risk of stage III or IV cancer (RR 0.50). Hormone receptor status appeared to be unaffected by the intervention [18].

In our cohort, there was no difference in oncologic outcomes of patients. Interestingly, patients in the MBS-group had a lower prevalence of relatively common BC pathologies such as DCIS and luminal-A type cancers. To our knowledge, this pattern has not been previously reported in the literature. These findings suggest that MBS may not only lower the incidence of BC but also influence the underlying tumor biology. Whether this reflects a delay in tumor progression, suppression of hormone-responsive pathways, or a change in immune and metabolic tumor microenvironments remains unclear. However, the relatively small cohort size and the retrospective design of this study limit our ability to draw definitive conclusions.

We hypothesize that the lack of significant differences in oncologic outcomes may be attributed to the lower prevalence of these subtypes in the MBS group. These subtypes are typically associated with more favorable oncologic outcomes, and their significantly lower prevalence in the MBS-group may explain the absence of differences in overall oncologic outcomes. Despite this, we believe this probably presents the benefit of MBS on prevention of more common hormone-mediated BC pathologies. This again emphasizes the advantage and protective effect of MBS on BC evolution.

Several limitations should be acknowledged. The retrospective design introduces potential selection bias and limits control over confounding factors and under-reporting. The cohort size is modest and drawn from a single center, which limits generalizability. Premenopausal women were not excluded, and hormonal status (e.g., insulin resistance, estrogen levels) was not uniformly assessed. Additionally, the BMI remained significantly different between groups at the time of BC surgery. Despite these limitations, our study has notable strengths, including the novelty of the research question and the use of detailed clinical data.

Future research should focus on prospective, multicenter trials with larger sample sizes and biomarker-based analysis to better understand the protective mechanisms of MBS on BC. Specifically, evaluating hormonal profiles, adipokine levels, and metabolic parameters pre- and postoperatively could elucidate the biological changes contributing to altered BC risk and presentation. Understanding these mechanisms may help guide individualized cancer prevention strategies for patients with obesity.

## Conclusions

MBS may play a role in breast cancer pathogenesis. In the MBS-group, we showed lower rates of more common breast cancer pathologies such as DCIS and luminal-A reflecting a shift away from early-stage breast cancer. It may also suggest a reduced overall incidence of breast cancer, potentially influenced by metabolic and hormonal changes following MBS. In the MBS-group, there was a higher prevalence of luminal-B subtype which could be due to the low prevalence of frequent pathologies. Oncologic outcomes were comparable. Long-term and larger cohort studies are needed for further clarification.

## Data Availability

No datasets were generated or analysed during the current study.
